# Glucocorticoid discontinuation in eosinophilic granulomatosis with polyangiitis treated with mepolizumab: associations with tapering patterns and initiation timing

**DOI:** 10.3389/fimmu.2026.1848854

**Published:** 2026-06-01

**Authors:** Takashi Yamane, Ayaka Inoue, Noriaki Yasuda, Takahisa Ohnishi, Akira Hashiramoto

**Affiliations:** 1 Department of Rheumatology, Kakogawa Central City Hospital, Kakogawa, Japan; 2 Department of Biophysics, Kobe University Graduate School of Health Sciences, Kobe, Japan

**Keywords:** eosinophilic granulomatosis with polyangiitis, eosinophils, glucocorticoid discontinuation, glucocorticoids, mepolizumab

## Abstract

**Introduction:**

Eosinophilic granulomatosis with polyangiitis (EGPA) requires prolonged glucocorticoid (GC) therapy in many patients, leading to substantial treatment-related morbidity. Although accumulating evidence has demonstrated the GC-sparing effects of mepolizumab (MPZ) and increasing numbers of patients achieving GC reduction or discontinuation, the patterns of GC tapering and discontinuation, including the influence of MPZ initiation timing, remain unclear.

**Materials and methods:**

We conducted a retrospective cohort study of EGPA patients treated with MPZ at a single center in Japan. We evaluated time to GC discontinuation and temporal changes according to MPZ initiation year. The interval from GC initiation to MPZ initiation was also assessed. Early GC discontinuation was defined as GC-free status within 500 days based on the observed temporal distribution of events. Time-to-event outcomes were analyzed using Kaplan–Meier and Cox regression, and early GC-free status was assessed using logistic regression.

**Results:**

Thirty-six patients were included. During a median follow-up of 4.6 years, GC discontinuation was achieved in 31 patients (86.1%), including 20 (55.6%) who achieved early GC-free status. The median time from MPZ initiation to GC discontinuation was 452 days. Higher eosinophil counts at MPZ initiation were independently associated with GC discontinuation (HR 1.45, 95% CI 1.08–1.96), whereas the interval from GC initiation to MPZ initiation was not. MPZ initiation in more recent years was associated with earlier GC discontinuation (OR 6.00, 95% CI 1.54–23.44), reflecting faster GC tapering after MPZ initiation. Vasculitis Damage Index (VDI) increased in 4 patients (11.1%) and was infrequent among those achieving early GC-free status.

**Conclusions:**

GC discontinuation was achieved in a high proportion of EGPA patients treated with MPZ. Accelerated GC tapering after MPZ initiation was associated with shorter GC treatment duration without an apparent increase in organ damage.

## Introduction

Eosinophilic granulomatosis with polyangiitis (EGPA) is a rare systemic vasculitis characterized by eosinophilic inflammation and heterogeneous organ involvement (1). Interleukin-5 (IL-5) plays a central role in the differentiation, activation, and survival of eosinophils, which are key drivers of tissue inflammation and damage in EGPA. Current treatment strategies rely on glucocorticoids (GCs) as the cornerstone of therapy, with the addition of immunosuppressive agents in severe cases (2). However, prolonged GC exposure is associated with substantial morbidity. Notably, longer duration of GC use and disease relapse have been linked to increased Vasculitis Damage Index (VDI) scores, reflecting cumulative and irreversible organ damage (3). Therefore, achieving GC tapering and discontinuation while maintaining disease control represents a major therapeutic goal in EGPA (4).

The phase 3 MIRRA trial demonstrated that targeting IL-5 with mepolizumab (MPZ) effectively reduces disease activity and GC exposure, leading to its widespread adoption in clinical practice (5). Subsequent treatment recommendations support the use of MPZ in patients with relapsing or refractory EGPA without severe organ- or life-threatening manifestations, aiming to improve remission and reduce GC dependence (6). Furthermore, accumulating evidence suggests that a substantial proportion of patients treated with MPZ can achieve GC discontinuation, including findings from clinical trials and real-world cohorts (7, 8).

Despite these advances, the patterns of GC tapering and discontinuation in routine clinical practice remain incompletely understood. In particular, it is unclear how GC tapering strategies have evolved, which factors are associated with successful GC discontinuation, and whether the timing of MPZ initiation influences the likelihood of achieving GC-free status. Addressing these questions is clinically important for optimizing IL-5–targeted therapeutic strategies and minimizing long-term treatment-related damage.

Accordingly, this study aimed to characterize longitudinal patterns of GC tapering, explore factors associated with GC discontinuation, and evaluate the relationship between GC discontinuation and clinical outcomes, including disease relapse and organ damage, in patients with EGPA treated with MPZ.

## Materials and methods

### Study design and patients

We conducted a retrospective cohort study of patients with EGPA treated with MPZ at a single center in Japan. During the study period, all patients diagnosed with EGPA at our institution were screened, and those who initiated MPZ after its approval were included. We included all consecutive patients who were eligible for at least 48 weeks at the time of data cutoff (October 2025). Patients were excluded if clinical data were unavailable or if follow-up was insufficient due to relocation or recent treatment initiation. EGPA was diagnosed based on established classification criteria, and clinical data were extracted from medical records. The 1990 American College of Rheumatology (ACR) criteria were primarily applied to patients diagnosed before 2022, whereas the 2022 ACR/European Alliance of Associations for Rheumatology (EULAR) classification criteria were mainly used for patients diagnosed thereafter (9, 10).

### Treatment and GC tapering

MPZ was initiated in patients with persistent or worsening EGPA-related disease manifestations despite standard therapy, including vasculitic features corresponding to the Birmingham Vasculitis Activity Score (BVAS) items as well as eosinophilic inflammatory symptoms such as asthma or upper airway disease. Additional considerations included increasing eosinophil counts or difficulty in tapering or discontinuing GCs. MPZ was initiated at a dose of 300 mg administered subcutaneously every 4 weeks. GC tapering was generally performed when disease-related symptoms were stable or absent and eosinophil counts were within the normal range, with the goal of eventual GC discontinuation. In addition, tapering was undertaken when there was no clinical evidence of worsening disease activity, including manifestations corresponding to BVAS items. GC dose reductions were typically performed in approximately 10% decrements, although tapering schedules were individualized. Decisions regarding GC tapering and the timing of MPZ initiation were made through shared decision-making between patients and treating physicians.

### Data collection

Demographic data included age and sex. Clinical manifestations at disease onset were recorded, including pulmonary, neurological, and other organ involvement. Disease severity was assessed using the Five-Factor Score (FFS) at disease onset and BVAS at MPZ initiation. Laboratory data included antineutrophil cytoplasmic antibody (ANCA) status and eosinophil counts at disease onset and at MPZ initiation. GC doses were recorded at disease onset, at MPZ initiation, and at the last follow-up visit. The interval from GC initiation to MPZ initiation was calculated for each patient and evaluated as a clinical variable. Concomitant immunosuppressant (IS) use at MPZ initiation was also recorded. Long-term organ damage was assessed using the VDI at MPZ initiation and at the last visit. Safety events during follow-up were also collected.

### Outcomes

The primary outcome was the time from MPZ initiation to first GC discontinuation. GC-free status was defined as the first complete discontinuation of GC. Secondary outcomes included early GC discontinuation, total duration of GC treatment, changes in organ damage assessed by the VDI, a validated instrument used to assess irreversible organ damage resulting from vasculitis or its treatment (11), and safety outcomes during follow-up. Early GC discontinuation was defined as GC-free status achieved within 500 days after MPZ initiation. This threshold was selected to reflect the temporal pattern of GC discontinuation observed in the cohort, including a higher rate of discontinuation during the early phase and the overall distribution of time to GC discontinuation. Sensitivity analyses were performed using alternative cut-off points (365 and 730 days) to assess the robustness of this definition. Relapse was defined as the recurrence or worsening of EGPA-related clinical manifestations, including those corresponding to BVAS items, requiring treatment escalation such as GC reintroduction or dose increase.

### Statistical analysis

Continuous variables are presented as median and interquartile range (IQR), and categorical variables as number (percentage). Time to GC discontinuation was analyzed using Kaplan–Meier methods. Temporal patterns of GC discontinuation were further evaluated using Nelson–Aalen cumulative hazard analysis. Factors associated with GC discontinuation were assessed using Cox proportional hazards regression. The proportional hazards assumption was evaluated using graphical methods and formally tested using time-dependent covariates, and no significant violations were observed (p > 0.05 for all variables). Given the limited number of events, the number of variables included in the multivariable logistic regression model for early GC discontinuation was restricted to avoid overfitting. Variable selection was based on both statistical significance in univariable analyses and clinical relevance. Because the survival curves suggested time-dependent changes in the rate of GC discontinuation, early GC discontinuation within 500 days was additionally analyzed using logistic regression. Correlations between clinical variables were assessed using Spearman’s correlation coefficient. For correlation analyses, linear regression lines with 95% confidence intervals were estimated using ordinary least squares. All analyses were exploratory, and no adjustments were made for multiple testing. A two-sided p value < 0.05 was considered statistically significant. Statistical analyses were performed using SPSS version 26 (IBM Corp., Armonk, NY, USA). This study was approved by the Ethics Committee of Kakogawa Central City Hospital (approval number: 2022-26) and was conducted in accordance with the Declaration of Helsinki.

## Results

### Patient characteristics

**Figure 1:**
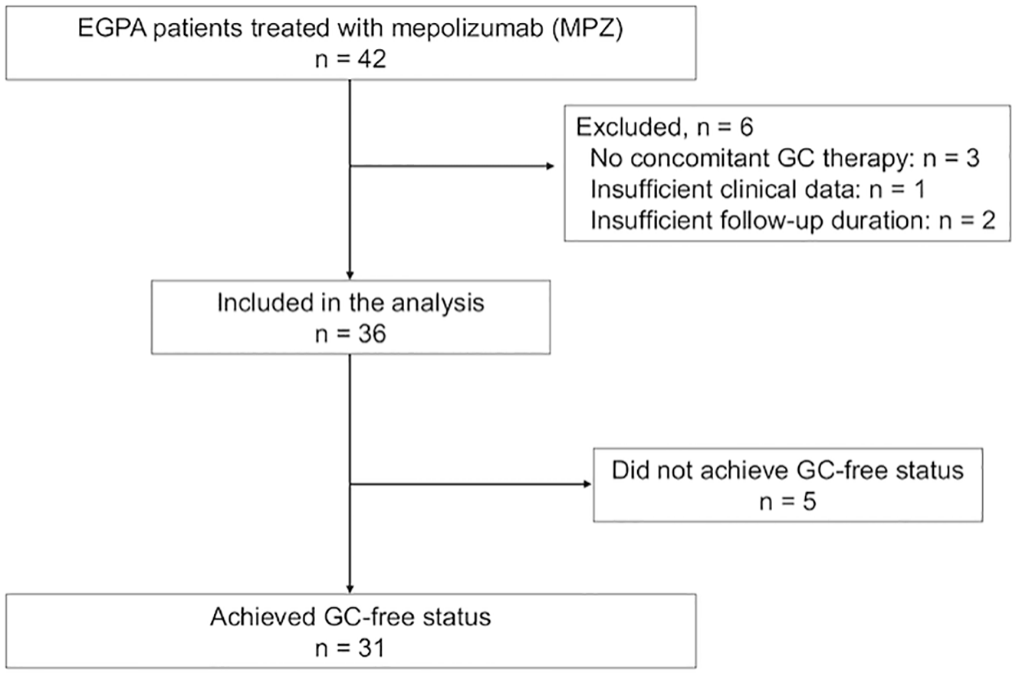
Study flow diagram. Of 42 patients with EGPA treated with MPZ, 6 were excluded because of no concomitant GC therapy, insufficient clinical data, or insufficient follow-up duration. The final analysis included 36 patients, of whom 31 achieved GC-free status.

A total of 42 patients with EGPA initiated MPZ during the study period. During the same period, an additional 3 patients with EGPA were managed without MPZ due to stable disease with low activity, maintenance on very low-dose GCs, or difficulty attending frequent hospital visits required for MPZ administration. Of the 42 patients who initiated MPZ, 6 were excluded due to no concomitant GC therapy (n = 2), insufficient clinical data (n = 2), or insufficient follow-up duration (n = 2), leaving 36 patients for the final analysis (**Figure 1**).

The median age at disease onset was 57 years, and 17 patients (47.2%) were female. ANCA positivity was observed in 11 patients (30.6%). At disease onset, the median eosinophil count was 7,056/µL, and most patients exhibited multisystem involvement, including neurological, pulmonary, and cardiovascular manifestations. The median FFS at onset was 1.0, with a distribution of 0 in 11 patients, 1 in 17 patients, 2 in 6 patients, and 3 in 2 patients. At MPZ initiation, the median BVAS was 3.0 (IQR 0.8–4.0). The median time from GC initiation to MPZ initiation was 411 days. At MPZ initiation, patients were receiving a median GC dose of 6.5 mg/day, and concomitant ISs were used in 17 patients (47.2%) (**Table 1**).

**Table 1:** Baseline characteristics, treatment features, and GC related outcomes of the overall cohort (n = 36).

Variable	Value
Patient characteristics at disease onset	
Female sex	17 (47.2)
Age at disease onset, years	57 (49-65)
ANCA positivity	11 (30.6)
Eosinophil count at disease onset,/µL	7056 (3096-16121)
Neurological involvement	26 (72.2)
Pulmonary infiltrates	16 (44.4)
Cardiovascular involvement	11 (30.5)
Renal involvement	5 (13.9)
Gastrointestinal involvement	9 (25.0)
Five-Factor Score (FFS) at onset	1.0 (0.0-1.8)
History of methylprednisolone pulse therapy	15 (41.7)
Initial GC dose at disease onset, mg/day	40 (30-50)
Treatment characteristics at MPZ initiation	
Time from GC initiation to MPZ initiation, days	411 (31-1270)
Eosinophil count at MPZ initiation,/µL	437 (269-1118)
BVAS at MPZ initiation	3.0 (0.8–4.0)
GC dose at MPZ initiation, mg/day	6.5 (3.6-16.9)
Concomitant IS use at MPZ initiation	17 (47.2)
GC-related outcomes during follow-up	
Achievement of GC-free status	31 (86.1)
Early GC-free status (within 500 days)	20 (55.6)
Time from MPZ initiation to GC discontinuation, days	452 (279-1157)
Total duration of GC therapy, days	1096 (486-2631)

Data are presented as median (interquartile range) or number (%), as appropriate. Early GC-free status was defined as GC discontinuation within 500 days after MPZ initiation. Patients included in the analysis had sufficient follow-up duration at the time of data cutoff. GC, glucocorticoid; MPZ, mepolizumab; IS, immunosuppressant; FFS, Five-Factor Score; BVAS, Birmingham vasculitis activity score; ANCA, antineutrophil cytoplasmic antibody.

### GC discontinuation outcomes

**Figure 2:**
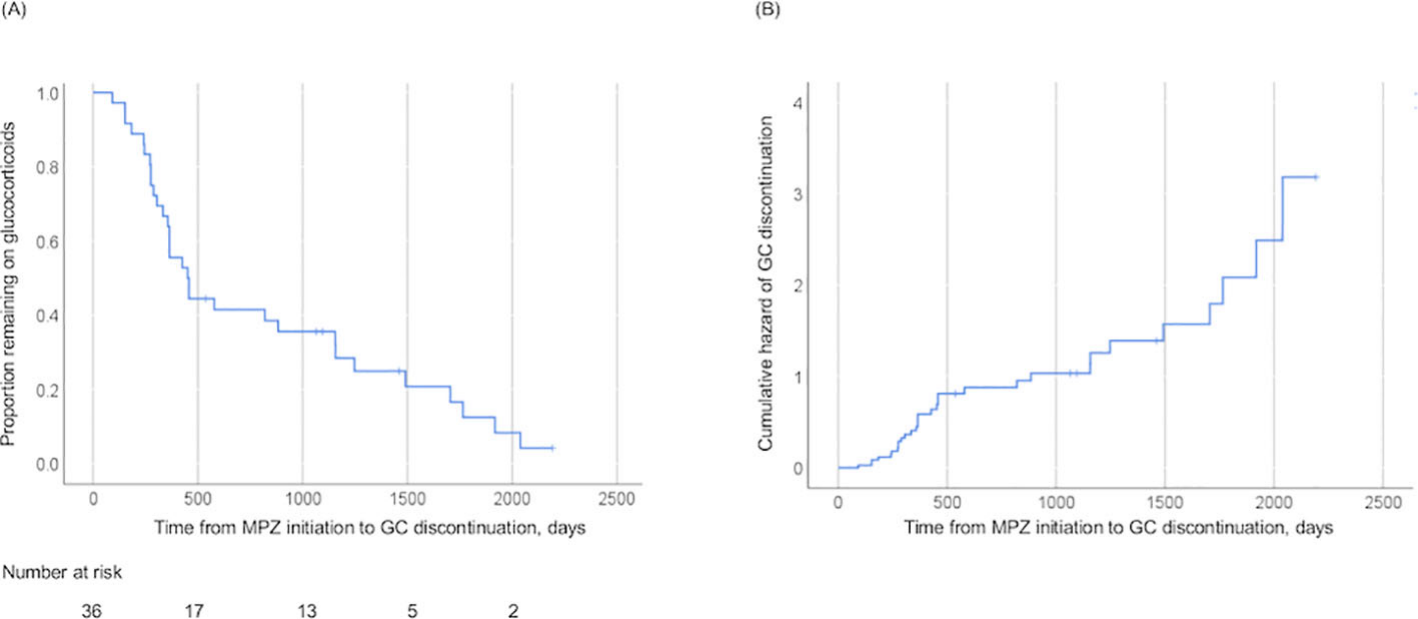
Time-to-event analysis of GC discontinuation. **(A)** Kaplan–Meier curve showing the proportion of patients remaining on GC after initiation of MPZ. A rapid decline is observed during the first 500 days, followed by a more gradual decrease thereafter. The vertical dashed line indicates 500 days, the threshold used to define early GC discontinuation. Tick marks indicate censored observations. **(B)** Nelson–Aalen cumulative hazard curve for GC discontinuation. The cumulative hazard increases rapidly during the early phase after MPZ initiation and more gradually beyond approximately 500 days, indicating a higher rate of GC discontinuation in the early period. GC, glucocorticoid; MPZ, mepolizumab.

The median time from MPZ initiation to GC discontinuation was 452 days. Kaplan–Meier analysis demonstrated a rapid decline in GC continuation during the first 500 days after MPZ initiation, followed by a more gradual decrease thereafter (**Figure 2A**). Consistently, the cumulative hazard curve showed a more rapid increase in GC discontinuation during the early phase, with a slower increase beyond approximately 500 days (**Figure 2B**). Early GC discontinuation was defined as GC-free status within 500 days, as described in the Methods section. Among these patients, 20 (55.6%) achieved early GC-free status.

In univariable Cox proportional hazards analyses, later year of MPZ initiation and higher eosinophil counts at MPZ initiation were significantly associated with a higher likelihood of GC discontinuation. In contrast, the interval from GC initiation to MPZ initiation was not significantly associated with GC discontinuation.

In the multivariable Cox model including these two variables, higher eosinophil counts at MPZ initiation remained independently associated with GC discontinuation (hazard ratio [HR] 1.45, 95% confidence interval [CI] 1.08–1.96; p = 0.014), whereas the association with year of MPZ initiation was attenuated and no longer statistically significant (HR 1.23, 95% CI 0.99–1.53; p = 0.066) (**Table 2**).

**Table 2:** Cox proportional hazards analysis of factors associated with GC discontinuation.

Variable	Univariable HR (95%CI)	P	Multivariable HR (95%CI)	P
Female sex	1.99 (0.95-4.16)	0.068	–	–
Age at disease onset, years	1.01 (0.98-1.04)	0.485	–	–
ANCA positivity	1.35 (0.63-2.88)	0.444	–	–
Eosinophil count at disease onset, per 1.0 ×10^9^/L	1.03 (0.98-1.07)	0.231	–	–
Five-Factor Score (FFS) at onset	0.79 (0.51-1.24)	0.643	–	–
Nasal involvement	0.55 (0.26-1.17)	0.122	–	–
Neurological involvement	2.07 (0.88-4.84)	0.095	–	–
Pulmonary infiltrates	1.32 (0.62-2.80)	0.466	–	–
Cardiovascular involvement	0.68 (0.30-1.50)	0.333	–	–
Renal involvement	0.62 (0.21-1.81)	0.384	–	–
Gastrointestinal involvement	0.60 (0.25-1.41)	0.236	–	–
Skin involvement	0.76 (0.37-1.59)	0.468	–	–
History of glucocorticoid pulse therapy	0.55 (0.26-1.15)	0.114	–	–
Initial daily GC dose, mg/day	0.98 (0.96-1.01)	0.144	–	–
Time from GC initiation to MPZ initiation, days	1.00 (1.00-1.00)	0.333	–	–
Year of MPZ initiation	1.28 (1.02-1.58)	0.030	1.23 (0.99-1.53)	0.066
Eosinophil count at MPZ initiation, per 1.0 ×10^9^/L	1.52 (1.14-2.03)	0.004	1.45 (1.08-1.96)	0.014
GC dose at MPZ initiation, mg/day	1.01 (0.98-1.04)	0.605	–	–
Concomitant IS use at MPZ initiation	0.52 (0.23-1.17)	0.116	–	–

Hazard ratios (HRs) are shown with 95% confidence intervals (CIs). HRs >1.0 indicate a higher likelihood of GC discontinuation. Eosinophil counts were scaled per 1.0 ×10^9^/L for regression analyses. The interval from GC initiation to MPZ initiation was analyzed per 100-day increase. Variables with a p value < 0.05 in univariable analyses were entered into the multivariable model, which was limited to two covariates to avoid overfitting. GC, glucocorticoid; MPZ, mepolizumab; IS, immunosuppressant; ANCA, antineutrophil cytoplasmic antibody; FFS, Five-Factor Score.

Factors associated with early GC-free status were examined using logistic regression analysis. In univariable analysis, gastrointestinal involvement was significantly associated with a lower likelihood of early GC-free status, whereas later year of MPZ initiation was significantly associated with a higher likelihood of early GC-free status.

In the multivariable model including these two variables, later year of MPZ initiation remained independently associated with early GC-free status (odds ratio [OR] 6.00, 95% confidence interval [CI] 1.54–23.44; p = 0.010), whereas gastrointestinal involvement showed a consistent but borderline association (OR 0.03, 95% CI 0.001–1.03; p = 0.052) (**Table 3**).

**Table 3:** Logistic regression analysis of factors associated with early GC discontinuation (within 500 days after MPZ initiation).

Variable	Univariable	P	Multivariable	P
Female sex	3.26 (0.67-16.02)	0.146	–	–
Age at disease onset, years	1.02 (0.98-1.07)	0.360	–	–
ANCA positivity	0.30 (0.06-1.51)	0.144	–	–
Eosinophil count at disease onset, per 1.0 ×10^9^/L	1.04 (0.94-1.16)	0.467	–	–
FFS at onset	0.44 (0.18-1.11)	0.081	–	–
Nasal involvement	1.20 (0.27-5.25)	0.809	–	–
Neurological involvement	0.31 (0.05-1.75)	0.185	–	–
Pulmonary infiltrates	0.98 (0.22-4.31)	0.981	–	–
Cardiovascular involvement	0.58 (0.12-2.87)	0.507	–	–
Renal involvement	0.14 (0.01-1.56)	0.110	–	–
Gastrointestinal involvement	0.13 (0.02-0.88)	0.036	0.03 (0.001-1.03)	0.052
Skin involvement	0.25 (0.05-1.16)	0.077	–	–
History of glucocorticoid pulse therapy	2.22 (0.50-9.99)	0.295	–	–
Initial daily GC dose, mg/day	0.96 (0.91-1.02)	0.151	–	–
Year of MPZ initiation	3.68 (1.51-8.93)	0.004	6.00 (1.54-23.44)	0.010
Time from GC initiation to MPZ initiation, days	1.03 (0.97-1.10)	0.262	–	–
Eosinophil count at MPZ initiation, per 1.0 ×10^9^/L	1.78 (0.65-4.85)	0.266	–	–
GC dose at MPZ initiation, mg/day	1.02 (0.96-1.08)	0.600	–	–
Concomitant IS use at MPZ initiation	0.20 (0.04-1.01)	0.052	–	–

Odds ratios (ORs) are shown with 95% confidence intervals (CIs). Early GC-free status was defined as complete GC discontinuation within 500 days after MPZ initiation. Variables with a p value < 0.05 in univariable analyses were entered into the multivariable model, which was limited to two covariates to avoid overfitting. GC, glucocorticoid; MPZ, mepolizumab; IS, immunosuppressant; ANCA, antineutrophil cytoplasmic antibody; FFS, Five-Factor Score.

**Figure 3:**
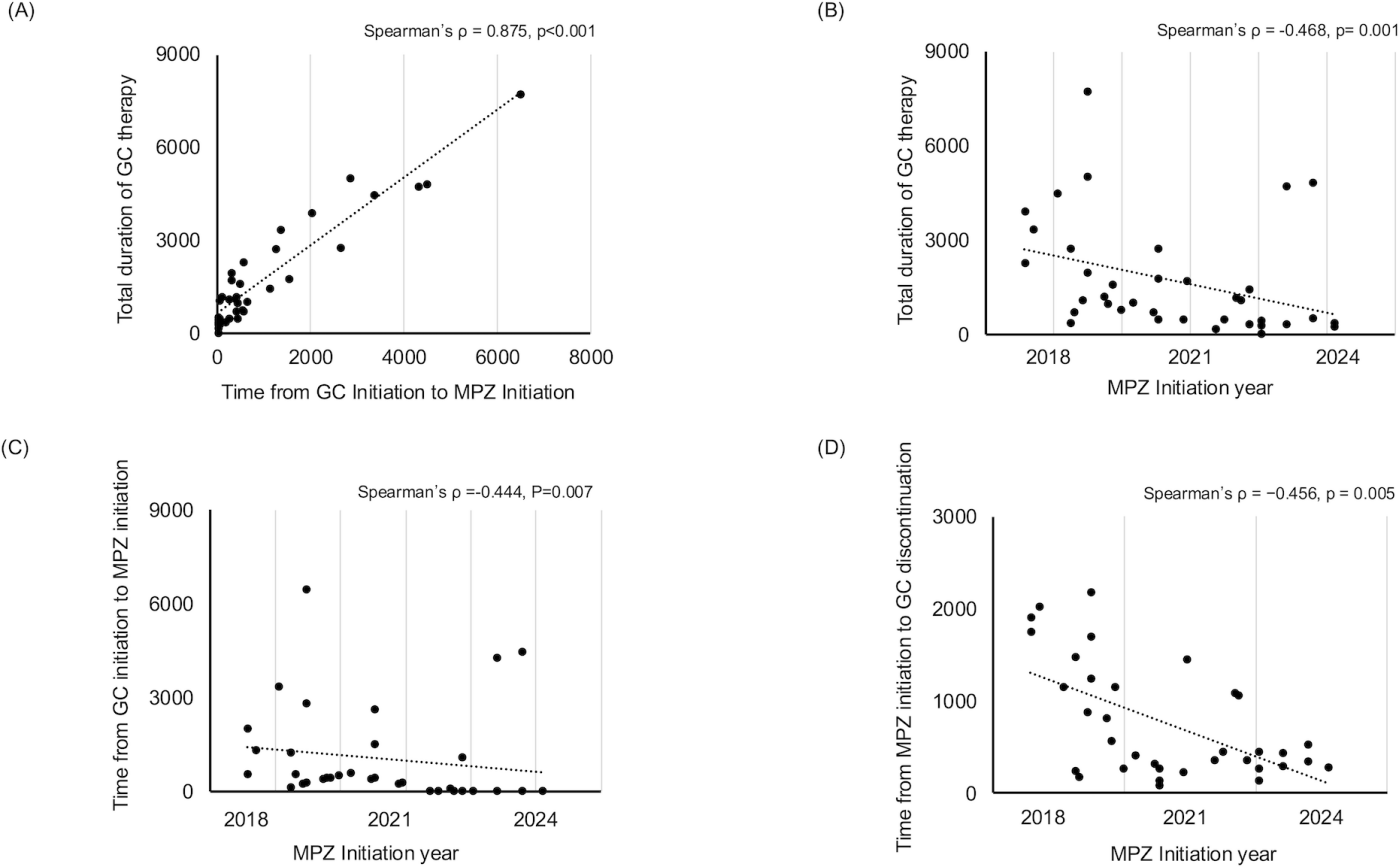
Correlations between timing of MPZ initiation and GC therapy duration. **(A)** Time from GC initiation to MPZ initiation versus total GC therapy duration. **(B)** Year of MPZ initiation versus total GC therapy duration. **(C)** Year of MPZ initiation versus time from GC initiation to MPZ initiation. **(D)** Year of MPZ initiation versus time from MPZ initiation to GC discontinuation. Solid lines indicate fitted linear regression lines, and dashed lines indicate 95% confidence intervals of the fitted values. Each dot represents an individual patient. Correlations were assessed using Spearman’s rank correlation coefficient. GC, glucocorticoid; MPZ, mepolizumab.

Correlation analyses demonstrated that the time from GC initiation to MPZ initiation was positively correlated with the total duration of GC therapy (Spearman’s ρ = 0.875, p < 0.001), indicating that longer intervals before MPZ initiation were associated with prolonged GC treatment duration (**Figure 3A**).

In contrast, the year of MPZ initiation was negatively correlated with total GC treatment duration (ρ = −0.468, p = 0.001) (**Figure 3B**). The year of MPZ initiation was also negatively correlated with the time from GC initiation to MPZ initiation (ρ = −0.444, p = 0.007), suggesting a modest trend toward earlier MPZ initiation in more recent years (**Figure 3C**). Notably, a negative correlation was observed between the year of MPZ initiation and the time from MPZ initiation to GC discontinuation (ρ = −0.456, p = 0.005) (**Figure 3D**), indicating that GC tapering after MPZ initiation has become progressively faster in more recent years.

At MPZ initiation, which occurred a median of 411 days after GC initiation, organ damage was present in 13 patients (36.1%). During follow-up, an increase in VDI score (ΔVDI > 0) was observed in 4 patients (11.1%) in the overall cohort. Among patients who achieved GC-free status, VDI increased in 4 patients (12.9%), whereas only one patient (5.0%) showed an increase among those who achieved early GC-free status. The median duration from MPZ initiation to the last follow-up visit was 4.6 years in the overall cohort, 4.7 years in GC-free patients, and 3.3 years in early GC-free patients (**Table 4**).

**Table 4:** Changes in Vasculitis Damage Index (VDI) from MPZ initiation to last follow-up.

Outcome	VDI at MPZ initiation			VDI at last visit			ΔVDI
	0	1	≧2	0	1	≧2	>0
Whole cohort (n=36)	23 (63.8)	11 (30.6)	2 (5.6)	22 (61.1)	9 (25.0)	5 (13.8)	4 (11.1)
GC-free cases (n=31)	–	–	–	19 (61.3)	8 (25.8)	2 (6.5)	4 (12.9)
Early GC-free cases (n=20)	–	–	–	12 (60.0)	7 (35.0)	1 (5.0)	1 (5.0)

Data are presented as number (percentage). ΔVDI > 0 indicates an increase in the VDI score from MPZ initiation to the last follow-up. Early GC-free status was defined as GC discontinuation within 500 days after MPZ initiation. VDI, Vasculitis Damage Index; MPZ, mepolizumab; GC, glucocorticoid.

During follow-up, one patient among the 31 who achieved GC-free status experienced a minor relapse requiring reintroduction of GC. MPZ was continued, and GC was subsequently tapered again, with the patient receiving 0.5 mg/day at the last follow-up. Five patients had not achieved GC discontinuation at the time of data cutoff. Of these, one patient was switched to benralizumab and was censored at that time point, while the remaining four patients continued gradual GC tapering.

Notably, no patients discontinued MPZ due to adverse events or inadequate vasculitis control.

## Discussion

In this retrospective cohort study of patients with EGPA treated with MPZ, GC discontinuation was achieved in the majority of patients (86%), and a substantial proportion achieved early GC-free status within 500 days. Importantly, GC discontinuation was not associated with the interval from GC initiation to MPZ initiation, suggesting that successful GC withdrawal is achievable irrespective of the timing of MPZ initiation.

Instead, MPZ initiation in more recent years was consistently associated with earlier GC discontinuation. However, this temporal association may also reflect changes in clinical practice over time, including increasing physician experience, evolving treatment strategies, and a greater tendency toward more aggressive GC tapering or earlier use of biologics in recent years, rather than a direct effect of tapering strategy itself, and should therefore be interpreted as an association rather than a causal relationship. Nevertheless, correlation analyses further indicated that this temporal effect was primarily driven by accelerated GC tapering after MPZ initiation rather than by earlier initiation of MPZ itself. These findings suggest that evolving tapering strategies in clinical practice may play a key role in reducing GC exposure. Although the definition of early GC discontinuation was based on the observed temporal pattern of GC tapering, sensitivity analyses using alternative cut-off points yielded consistent results, supporting the robustness of this finding. Notably, the interval from GC initiation to MPZ initiation showed only modest temporal shortening, whereas GC tapering after MPZ initiation became progressively more rapid, indicating that post-treatment tapering strategies were the primary contributors to shorter GC treatment duration. This may reflect that treatment response to MPZ facilitates GC tapering irrespective of when MPZ is initiated, provided that disease control is achieved.

Our findings are consistent with previous studies demonstrating the GC-sparing effects of MPZ. Prior clinical trials, including the MIRRA study, have shown that MPZ improves disease control and enables substantial GC reduction, particularly in patients with higher baseline eosinophil counts (4). In line with these findings, higher eosinophil counts at MPZ initiation were associated with GC discontinuation in our study. In contrast, gastrointestinal involvement was associated with a lower likelihood of early GC-free status in univariable analysis, although this association did not remain statistically significant after adjustment. Gastrointestinal manifestations in EGPA are generally considered markers of more severe disease (12), and this finding may reflect greater caution in GC tapering among such patients in clinical practice.

Subsequent studies, including our prior Japanese cohort, have consistently demonstrated the GC-sparing effects of MPZ, with many patients achieving GC-free status (7, 8, 13). These findings are in line with our results, which also showed a high rate of GC discontinuation.

In addition, previous studies have reported that MPZ reduces GC doses and improves survival, with clinical benefits observed regardless of concomitant immunosuppressant use or disease duration (14, 15). This supports the broad applicability of MPZ in real-world clinical practice. Notably, recent Japanese post-marketing surveillance data showed that patients receiving long-term MPZ with higher average GC doses experienced more adverse events and higher relapse rates (16), highlighting the clinical importance of reducing GC exposure.

Moreover, sustained remission with GC withdrawal has been reported not only with MPZ but also with benralizumab, suggesting that timely implementation of IL-5–targeted biologic therapy, rather than the selection of a specific agent, may be associated with reduced GC exposure in EGPA (17).

Importantly, no clear increase in organ damage was observed in association with accelerated GC tapering, although the number of patients with worsening VDI was small. Only a small proportion of patients showed increases in VDI, particularly among those achieving early GC-free status. However, these findings should be interpreted with caution given the limited number of events, differences in follow-up duration, and the lack of adjustment for baseline VDI values. Given that cumulative GC exposure and disease activity are both associated with irreversible organ damage in ANCA-associated vasculitis (3, 18), and that such damage correlates with impaired health-related quality of life (19), reducing GC exposure while maintaining disease control is clinically important. Despite this recognition, a recent report indicates that most patients with EGPA continue to receive prolonged GC therapy, while the use of biologic agents remains limited, resulting in a substantial treatment burden and high healthcare resource utilization (20). In addition, relapse requiring GC reintroduction was rare and manageable under continued MPZ therapy, further supporting the clinical feasibility of GC discontinuation. Although the median FFS was relatively low, a substantial proportion of patients had higher prognostic risk, suggesting that the cohort was not limited to patients with low disease burden.

This study has several limitations, including its single-center retrospective design, limited sample size, and lack of a control group, which may limit generalizability and preclude causal inference. In addition, patients who had not yet reached 48 weeks of MPZ treatment at the time of data cutoff were excluded, which may have influenced the observed GC discontinuation rate. Despite the small number of excluded patients, potential selection bias cannot be entirely ruled out.

Importantly, treatment decisions, such as the timing of MPZ initiation and GC tapering, were not standardized and were made at the discretion of the treating physicians. These decisions likely reflect evolving clinical expertise, changes in guidelines, and improvements in supportive care over time, and therefore should not be considered fully independent or objective variables. Accordingly, the observed temporal trends should be interpreted as associations rather than direct evidence of a causal relationship between specific tapering strategies and outcomes and may also reflect temporal changes in clinical practice as a potential source of bias.

Furthermore, disease activity was not systematically assessed using standardized instruments such as BVAS at all time points. While we evaluated disease control based on clinical manifestations and treatment changes, this may limit the precision of assessing safety during GC tapering.

Additionally, variations in treatment practices across regions and the unmeasured impact of concomitant ISs may further limit generalizability. Moreover, previous studies have highlighted substantial regional and demographic differences in ANCA-associated vasculitis populations, including the underrepresentation of non-White patients in clinical trials, which may affect the generalizability of findings across different healthcare settings (21). In addition, given the relatively low median FFS at disease onset, the applicability of our findings to patients with more severe EGPA may be limited.

Nevertheless, the consistency of findings across multiple analytical approaches (including sensitivity analyses) strengthens the validity of our conclusions.

In conclusion, GC discontinuation is achievable in most patients with EGPA treated with MPZ. Accelerated GC tapering after MPZ initiation was associated with shorter GC treatment duration in real-world clinical practice. However, the relative contributions of tapering strategies and the timing of MPZ initiation cannot be determined from this study. Accelerated GC tapering may also be associated with reduced treatment-related and disease-related organ damage.

## Data Availability

The raw data supporting the conclusions of this article will be made available by the authors, without undue reservation.
